# Shall Nurses Cease to Be Lay Women?—I

**Published:** 1891-09-05

**Authors:** 


### Passing Topics.

### SHALL NURSES CEASE TO BE LAY-WOMEN?—I.

Although a great deal has been uttered within recent years upon the subject of nursing, and the doings of the Association which claims to be particularly concerned in defining the status of nurses, have been freely criticised, we do not remember that the question at the head of this paper, and which seems to us of the first importance, has received any, or at all events adequate, attention.

The position taken up by the British Nurses' Association was very frankly stated at the outset, and has been set forth by the *Lancet* thus:—

"The founders of the new association clearly saw that the time had come to unite nurses in the membership of a legally constituted profession, and they also knew that the control of this profession must be kept solely in the hands of medical men and nurses. . . . It (the association) merely claims for medical men and nurses the bare right to manage the nursing profession without the interference of any of the lay public, however well intentioned."

This manifesto of professionalism was fully maintained at the inaugural meeting in St. George's Hall. The interference, even the co-operation, of the "laity" was summarily rejected. Not once but several times the audience was told that the Association was to be kept exclusively in the hands of the medical and nursing "professions," and that the control of nurses was altogether outside the sphere of laymen.

The foremost and avowed object of the Association is, therefore, to constitute nursing a profession, and we have to consider what will be the results if the pretension be made good. It would be manifest, even without what has gone before, that a nurse who has ceased to be a laywoman, and has become the member of a profession, will object to government by laymen, doctors included, who will, of course, be "laity" so far as the nursing "profession" is concerned. In this she will only imitate those who must of necessity be her exemplars—the doctors. Out of this fact arise several considerations, important to the nurses themselves, and even more important to hospital managers, hospital patients, and the medical profession. First, let us ask, what will be the effect upon hospital discipline? That there are already indications of what may be expected, few who are concerned with hospital work will deny. Nurses, as a body, are giving evidence that they are not insensible to the attentions of which they have been the subjects of late, and it is only natural they should welcome the notion of a recognised professional status for their calling, and that the more ambitious and, possibly, the less intelligent of them should draw conclusions not altogether justified by the premises. In this they are not so much in fault as the leaders, who reason from abstract propositions without so much as a reference to considerations they choose to ignore.

That the danger to discipline is real may be gathered from the following and other similar communications to which currency has been given in the organ of registration:—

"Sir," writes "Sister," who, be it remembered, is taking the pay of the hospital, "the certificated nurses of this hospital, nearly all of whom are 'registered,' are very grateful to you for your support of our attempt at reform in nursing matters. We believe our Matron, who advocates registration, knows more about the requirements of the nurses than the Governors of the hospital—gentlemen occupied in business and trade, who never enter the hospital, or know a Sister from a ward-maid"—dire ignorance indeed!—"and we have firmly made up our minds, with all due respect to the Committee and Governors, to register our certificates as we obtain them. If our independence savours of insubordination we do not intend it to do so, and feel sure we shall still try to perform our duties to the best of our ability."

We are aware it has long been the opinion of a minority of the medical profession, the junior and least informed section, that the one function of the "lay" authorities of hospitals is to finance them, but it argues little for the intelligence of a Sister that, because she has seldom to look beyond the Matron or the House Surgeon for her instructions, she should suppose that the higher authorities, representative of those by whose favour the organism exists, and possibly, by whose efforts it has been created, are either non-existent or useless.

(*To be continued.*) Agricola.

